# From neurobiological regulation to socio-ecological remodeling: a mini review of yoga interventions for adolescent smartphone addiction

**DOI:** 10.3389/fpsyg.2026.1782953

**Published:** 2026-06-02

**Authors:** Xiaohong An, Weibing Ye, Yichen Fan

**Affiliations:** 1 School of Physical Education and Health, Yili Normal University, Yining, China; 2 College of Physical Education and Health Sciences, Zhejiang Normal University, Jinhua, Zhejiang, China; 3 Institute of Sports Health and Nutrition, Beijing Competitor Sports Science & Tech. Co., Ltd, Beijing, China

**Keywords:** BDNF, embodied cognitive training, mind-body intervention, neurobiology, socio-ecological model

## Abstract

Adolescent smartphone addiction has emerged as a pervasive public health crisis, which often stems from a developmental imbalance between hypersensitive reward circuitry and immature executive control. While conventional interventions such as cognitive-behavioral therapy remain the standard of care, they are often constrained by a lack of holistic focus, failing to address the multifaceted physiological dysregulation and environmental triggers associated with addictive behaviors. This review synthesizes the neurobiological mechanisms of yoga, positioning it not merely as physical exercise but as a form of “embodied cognitive training” that bridges the gap between physiological regulation and psychological recovery. We examine how yoga may facilitate psychophysiological homeostasis through a dual pathway: a potential “bottom-up” regulation that attenuates HPA axis hyperactivation and enhances vagal tone, and a proposed “top-down” reinforcement of prefrontal connectivity to inhibit impulsive reward-seeking. At the molecular level, we discuss evidence suggesting that yoga-induced upregulation of BDNF and GABA may provide a potential neuroplastic substrate helpful for repairing compromised neural networks and restoring reward prediction error processing. However, recognizing that neuroplasticity depends on environmental context, we argue that sustainable recovery necessitates extending beyond individual neuroadaptation to a “Home-School-Community” synergistic model. By combining targeted neurobiological regulation with broad environmental support, this framework offers clinicians a practical strategy to help adolescents build cognitive resilience and regain control over their digital habits.

## 1 Introduction

Adolescent smartphone addiction, clinically often termed problematic smartphone use (PSU), has escalated into a pervasive global public health crisis. It is characterized by compulsive engagement, withdrawal symptoms, and functional impairment in daily life. A recent meta-analysis encompassing over 5,000 students revealed a prevalence rate of 41.93%, highlighting its strong associations with chronic stress, depression, and academic decline (Zhong et al., 2022). Longitudinal evidence further warns that such addiction may induce maladaptive structural and functional neuroadaptations during the critical plasticity window of adolescence (Wacks and Weinstein, 2021).

**Figure 1:**
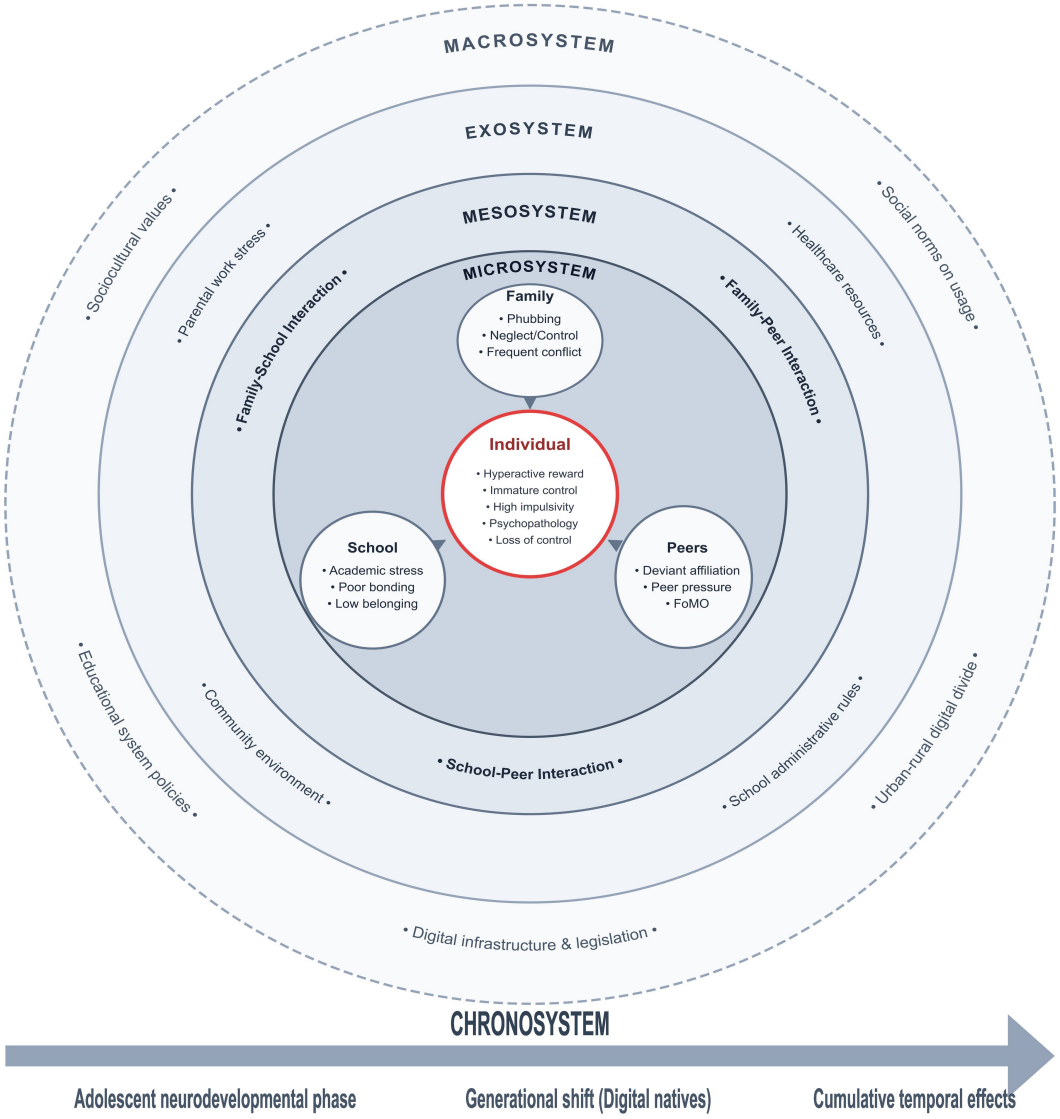
Social ecological model of adolescent smartphone addiction.

The etiology of this addiction is deeply rooted in the unique neurobiological milieu of adolescence, specifically a “developmental mismatch.” This window is marked by a dual-system imbalance: a heightened sensitivity of the reward system—anchored in the ventral tegmental area (VTA) and nucleus accumbens (NAc)—which becomes hyper-responsive to immediate social feedback such as “likes” and notifications (Mascia et al., 2020). Conversely, the executive control network, primarily the prefrontal cortex (PFC), remains structurally and functionally immature. This functional lag creates a vulnerability window wherein adolescents are particularly susceptible to pathological reward-seeking behaviors and impulsive feedback loops, a risk exacerbated by psychological distress (Stelly et al., 2020). To provide a clearer conceptual framing of this process, we have synthesized these multifaceted factors into an integrated Eco-Neurobiological conceptual flow chart (Figure 1). Grounded in the Social-Ecological Model (SEM), this diagram illustrates how external environmental triggers—particularly dysfunctions within the family and school microsystems—interact with individual psychological vulnerabilities. This psychosocial cascade ultimately funnels into the core neurobiological imbalances (stress hyperarousal, reward hijacking, and executive deficits), forming a maladaptive reinforcement loop that culminates in Smartphone Addiction.

While conventional interventions such as cognitive-behavioral therapy (CBT) remain the standard of care, recent evidence highlights their limitations in addressing the multifaceted nature of addiction, particularly the physiological dysregulation of stress and reward systems (Malinauskas and Malinauskiene, 2019; Chen et al., 2025). Consequently, academic focus has shifted toward mind–body interventions. Yoga, integrating physical postures, breath regulation, and meditation, has evolved into a form of “embodied cognitive training” (Brems, 2025; Li et al., 2025; Jagadambal, 2025). Emerging neuroimaging evidence suggests that yoga can modulate these dysregulated brain networks, offering a “bottom-up” physiological pathway to restore homeostasis.

However, neurobiological recovery does not occur in a vacuum. The socio-ecological model posits that health behaviors emerge from the interplay between individuals and their environments (Singh, 2024). We argue that while yoga provides the neuroplastic substrate for recovery (e.g., via BDNF upregulation), sustainable behavioral change necessitates a supportive environment to consolidate these neural gains. Therefore, effective intervention requires a paradigm shift from isolated individual treatments to a synergistic “Home-School-Community” model. This article aims to synthesize the neurobiological mechanisms of yoga and propose an integrated framework for promoting socio-ecological remodeling in the management of adolescent smartphone addiction.

## 2 Search strategy and selection criteria

To enhance methodological transparency and rigor, the literature search and study selection process for this Mini Review were structured as a narrative review. A comprehensive search was conducted using academic databases including PubMed, Web of Science, Scopus, and APA PsycINFO, alongside Google Scholar, for articles published up to November 30, 2025. To ensure reproducibility, the following Boolean search string was applied across titles, abstracts, and keywords: (“smartphone addiction” OR “problematic smartphone use” OR “mobile phone dependence” OR “internet addiction”) AND (“yoga” OR “yogic practice” OR “mind–body intervention” OR “mindfulness-based”).

Study inclusion was meticulously guided by the adapted PICOS criteria: focusing on adolescents and young adults aged 10 to 24 (Sawyer et al., 2018) participating in yoga-based mind–body interventions, with outcomes evaluating reductions in addiction severity and improvements in psychophysiological well-being. Crucially, given the nascent stage of research intersecting yoga and digital addiction, we adopted an inclusive approach. To capture a comprehensive socio-ecological snapshot, we included diverse designs ranging from rigorous Randomized Controlled Trials (RCTs) to pilot studies and cross-sectional analyses. Studies not peer-reviewed or published in non-English languages were excluded. Ultimately, 7 key empirical studies that met these rigorous criteria were selected and synthesized in this review (as summarized in Table 1). Importantly, these 7 studies were included not as an exhaustive systematic sample, but as core empirical evidence demonstrating the behavioral and psychological efficacy of yoga interventions for digital addiction. This clinical baseline justifies and provides the foundation for our subsequent theoretical exploration of the underlying neurobiological mechanisms and the proposed socio-ecological implementation model.

**Table 1:** Characteristics and key findings of included studies on yoga interventions for digital addiction.

Author (Year)	Study design	Participants	Intervention protocol	Control / comparator	Key findings
Thapliyal et al. (2025)	RCT	University students (*N* = 150)	F: Daily T1: Traditional yoga T2: 60 min/session D: 10 weeks	Normal daily routine	Significant reduction in smartphone addiction severity
Reshu et al. (2025)	Pilot RCT	High school students	F: Daily T1: School-based yoga T2: 25 min/session D: 3 months	Routine school activity	Improved internet resilience and concentration; reduced anxiety
Mona et al. (2024)	RCT	Adolescents (13–17 yrs)	F: Daily T1: Integrated yoga T2: 45 min/session D: 12 weeks	Attentional control group	Decrease in smartphone addiction scores, anxiety, depression, and total screen time
Rao et al. (2025)	Methodological study	Adolescents	F: 3 days/week T1: Integrated yoga T2: 40 min/session	Baseline	Reduced IGD symptoms and improved academic performance.
Putchavayala et al. (2022)	Single-arm pilot	Young adolescents	F: Daily T1: Specific yoga D: 6 weeks	Baseline (pre-intervention)	Reduction in addiction symptom scores post-intervention.
Pal et al. (2022)	Cross sectional	University students (*N* = 284)	F: 6 days/week T2: 90 min/session D: 30 months	Non-yoga practitioners	Lower SAS scores; fewer nocturnal smartphone checks.
Li et al. (2025)	Cross-sectional	Female college students (*N* = 554)	Mind–body interventions (including Yoga/Tai Chi)	N/A (Correlation)	Yoga/Tai Chi practitioners showed the highest mindfulness scores and lowest mobile phone addiction

F, Frequency; T1, Type; T2, Time; D, Duration; RCT, Randomized Controlled Trial; IYM, Integrated Yoga Module; SAS, Smartphone Addiction Scale; IGD, Internet Gaming Disorder.

## 3 The biopsychosocial and ecological context of addiction

Grounded in the Social Ecological Model, smartphone addiction in adolescents operates within a complex web of psychological and environmental factors rather than as an isolated phenomenon (Figure 1). At the individual core, vulnerability often stems from a neurodevelopmental mismatch—such as a hyperactive dopamine reward system coupled with immature prefrontal executive control (Chen et al., 2019; Brand et al., 2019). A bidirectional relationship exists between addiction and psychopathology: cross-sectional data demonstrate that anxiety, depression, and post-traumatic stress disorder (PTSD) not only predict higher addiction risk but also serve as maladaptive coping mechanisms that perpetuate excessive use (Kim et al., 2021; Cheng et al., 2024; Padmanabhanunni and Pretorius, 2025).

Furthermore, the microsystem plays a pivotal role. Research confirms that dysfunctional family dynamics—such as “phubbing,” frequent conflict, and negative parenting styles—significantly mediate adolescent addiction risks (Mun and Lee, 2021; Doo and Kim, 2022). Conversely, strong parent–child bonding acts as a protective buffer (Gong et al., 2022). Beyond the home, concurrent microsystem stressors, including high academic stress at school and peer-related pressures like deviant affiliation and the Fear of Missing Out (FoMO), further exacerbate individual vulnerabilities (Li et al., 2022; Yoo, 2024). These immediate environments are inextricably linked through the mesosystem (e.g., family-school interactions) and are heavily influenced by broader exosystemic and macrosystemic forces, such as parental work stress and digital sociocultural norms, all unfolding within the critical chronosystem of puberty (Hsieh et al., 2021). These findings underscore that while the symptoms are neurobiological, the triggers are often psychosocial and ecological. Consequently, effective interventions must extend beyond individual symptom management to address the broader environmental ecosystem.

## 4 Psychophysiological foundations of yoga: a step-wise mechanistic mapping

**Figure 2:**
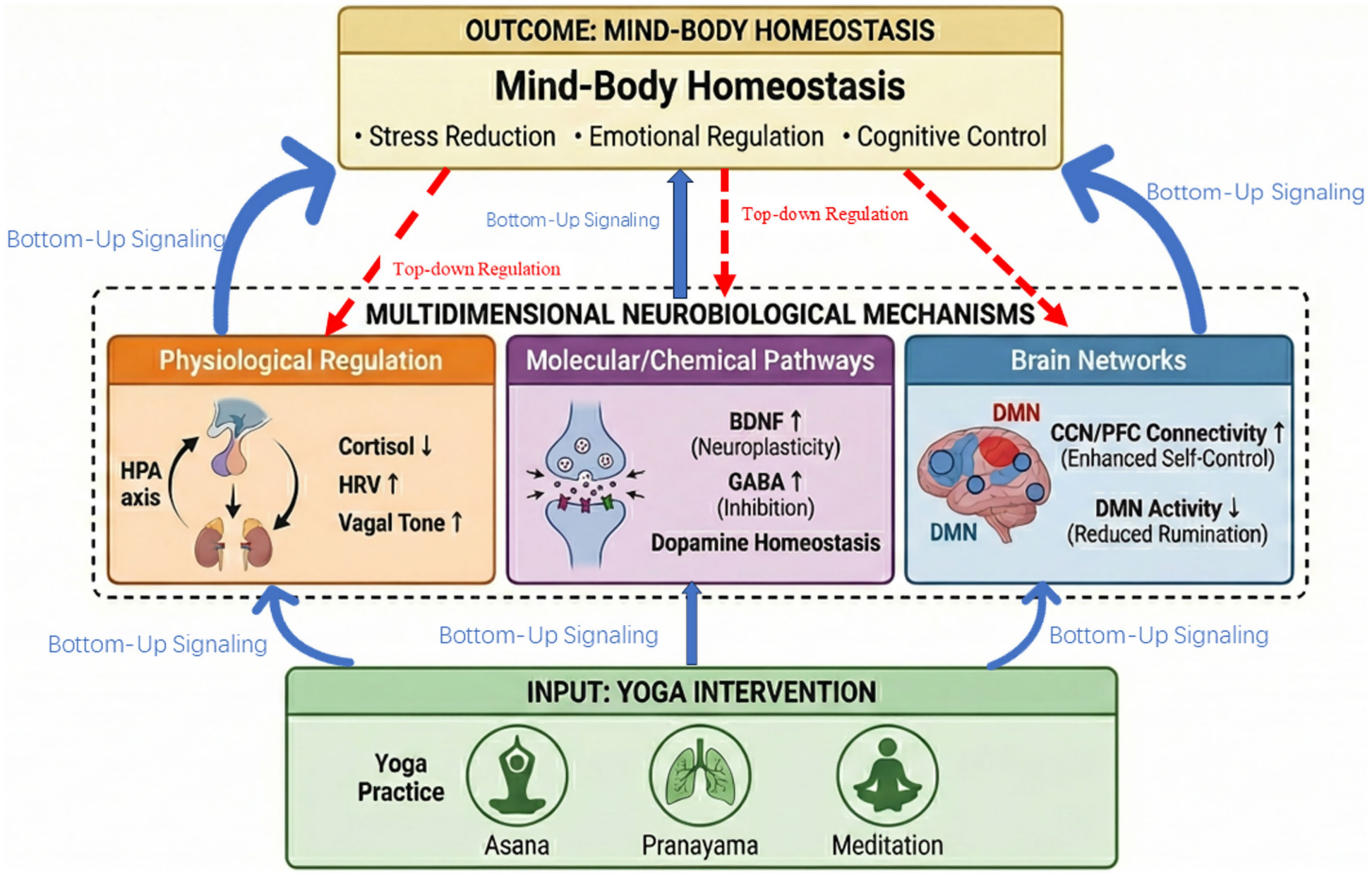
Neurobiological mechanisms of yoga promoting mind–body homeostasis.

As a multimodal mind–body practice integrating physical postures (Asanas), breath regulation (Pranayama), and meditation (Dhyana), yoga exerts health-promoting effects that extend far beyond non-specific placebo responses. Instead, it facilitates systemic remodeling ranging from molecular pathways to macroscopic neural networks (Voss et al., 2023; Venditti, 2025). Current translational evidence indicates that yoga fosters psychophysiological homeostasis through a dual mechanism: “bottom-up” physiological arousal regulation and “top-down” cognitive control enhancement (Voss et al., 2023; Brems, 2025; Figure 2).

### 4.1 Step 1: pranayama and autonomic regulation (targeting stress hyperarousal)

As the foundational step, controlled breathing (Pranayama) is hypothesized to acts as the primary physiological modulator, potentially stimulating the vagus nerve to downregulate HPA axis hyperactivation (Nagar and Betal, 2023). Chronic physiological dysregulation induced by stress underpins the maintenance of both psychosomatic disorders and addictive behaviors. Yoga interventions address this by counteracting the “fight-or-flight” response, primarily through the downregulation of hypothalamic–pituitary–adrenal (HPA) axis hyperactivation (Basu-Ray, 2021). Empirical studies suggest that regular practice may significantly attenuates serum cortisol levels, which could contribute to mitigating the suppression of hippocampal neurogenesis caused by chronic glucocorticoid exposure. Complementing this hormonal regulation, specific yogic breathing techniques—particularly those emphasizing a prolonged exhalation (e.g., 1:2 ratio)—directly stimulate the vagus nerve. This stimulation enhances Heart Rate Variability (HRV), a robust marker of parasympathetic dominance (Zou et al., 2018). Such autonomic flexibility functions as a physiological “brake,” potentially facilitating a rapid shift from stress states to restoration, thereby helping to inhibit impulsive behaviors.

### 4.2 Step 2: Dhyana and neurotransmitter dynamics (targeting reward hijacking)

Building upon physiological calmness, meditative practices (Dhyana) are thought to engage top-down cognitive control to target impulsivity and reward hijacking. Evidence suggests it may modulate dopamine and GABA dynamics to potentially assist in repairing reward prediction errors and enhance prefrontal inhibitory control over compulsive smartphone craving (Kirk et al., 2019).

Simultaneously, yoga addresses the dysregulated reward system. Unlike the transient, high-amplitude dopamine spikes triggered by digital stimuli, yoga fosters a sustained endogenous reward state through the synergistic release of endorphins and endocannabinoids (Sadhasivam et al., 2020). This mechanism is hypothesized to assist in repairing reward prediction errors, facilitating a neural shift away from dependency on high-intensity external stimuli toward a sensitivity to natural rewards (Kirk et al., 2019).

### 4.3 Step 3: Asanas and molecular neuroprotection (targeting executive deficits via BDNF)

Finally, the physical exertion of postures (Asanas) may target executive deficits via structural neuroplasticity. This practice is thought to stimulate muscle-brain crosstalk, potentially upregulating Brain-Derived Neurotrophic Factor (BDNF). As a cornerstone of neuroplasticity, BDNF governs synaptic growth and connectivity repair (Cahn et al., 2017). Yoga exerts neuroprotective effects through a specific pathway where muscle contractions during Asanas stimulate the secretion of myokines such as irisin, which subsequently induces BDNF expression in the hippocampus (Cahn et al., 2017). Clinical interventions in broader populations have documented that systematic yoga practice is associated with significant elevations in plasma BDNF levels (Sonawane et al., 2025). This molecular surge is theorized to provide a potential material substrate for repairing damaged synaptic connections, ultimately enhancing memory consolidation and cognitive flexibility in adolescents.

### 4.4 The dual regulatory framework: bottom-up and top-down processing

Synthesizing these mechanisms, yoga’s therapeutic efficacy is proposed to operate through a simultaneous dual regulatory framework (Figure 2). First, the bottom-up model (somatic-to-cognitive) is driven by Pranayama and Asanas, altering central processing via peripheral physiological changes. Slower respiration and physical postures stimulate vagal afferents, transmitting “safety signals” to the limbic system to dampen amygdala hyperactivity and HPA axis reactivity, thereby diminishing the physiological urge for digital stress-relief. Conversely, the top-down model (cognitive-to-somatic), engaged primarily through Dhyana, utilizes focused attention to activate the prefrontal cortex (PFC). This enhanced PFC connectivity is hypothesized to exert an inhibitory influence over the striatal reward circuitry (e.g., VTA-NAc), potentially restoring executive control to consciously override impulsive smartphone cravings. Together, these bidirectional pathways synchronize rehabilitation, addressing addiction as a systemic mind–body imbalance rather than isolated cognitive or physical deficits.

## 5 Current empirical evidence and methodological challenges

### 5.1 Efficacy of yoga interventions

Based on the shared neurobiological mechanisms of addiction, the efficacy of yoga in substance dependence provides a strong theoretical basis for its application in smartphone addiction. As summarized in Table 1, a growing body of empirical research has validated the efficacy of various yoga protocols in mitigating digital addiction. Studies range from cross-sectional surveys to Randomized Controlled Trials (RCTs), targeting diverse populations from high school adolescents to university students. The intervention protocols typically employ an “Integrated Yoga” approach (combining Asana, Pranayama, and meditation) with varying durations (6 weeks to 30 months) and frequencies (daily to 3 days/week). Despite heterogeneity in methodology, the findings consistently demonstrate that yoga intervention significantly reduces addiction severity scores (e.g., SAS-SV, PUMP) and ameliorates comorbid psychopathology, including anxiety, depression, and sleep disturbances. Notably, recent RCTs highlight dose-dependent benefits, where regular practice correlates with improved “Internet Resilience” and cognitive concentration.

### 5.2 The ecological gap: limitations of current RCTs

Despite the documented efficacy of yoga interventions, current research faces a critical limitation: a lack of ecological validity. The majority of existing Randomized Controlled Trials (RCTs) are conducted in strictly controlled environments, such as schools or laboratories (Mona et al., 2024; Thapliyal et al., 2025). While this design ensures internal validity, it fails to account for the home environment, which is often the primary setting for addictive behaviors and digital conflict. Adolescent smartphone addiction is rarely an isolated pathology; rather, it frequently manifests as a symptom of broader family dysfunction. Recent evidence highlights that maladaptive parental behaviors, such as “phubbing” (parental phone snubbing) and a lack of meaningful parent–child interaction, are significant predictors of adolescent addiction (Mun and Lee, 2021; Doo and Kim, 2022). Consequently, interventions that target the adolescent in isolation—without addressing family dynamics—are likely to yield diminished long-term sustainability. Crucially, a temporal asymmetry exists between neurobiological recovery and digital reward processing. While evidence confirms that yoga upregulates BDNF and modulates GABA, these neuroplastic changes typically require months of sustained intervention to stabilize. In the short term, this gradual physiological remodeling cannot compete with the ‘high-dopamine immediate feedback’ loops inherent to smartphone addiction. Consequently, sustainable recovery requires more than isolated physiological regulation. By actively embedding yoga into daily family and school routines, we can effectively bridge the gap between slow neural adaptations and the adolescent’s need for immediate behavioral rewards. To overcome these limitations, future research must shift from isolated individual interventions to a systemic approach.

### 5.3 Methodological limitations of current evidence

While the synthesized findings demonstrate promising efficacy, they must be interpreted with caution due to several pervasive methodological limitations in the existing literature. First, a majority of the included studies rely on relatively small sample sizes or single-arm pilot designs. This inherently limits the statistical power of the results and restricts the generalizability of the findings to broader adolescent populations.

Second, there is substantial heterogeneity in the intervention protocols. The included studies encompass widely varying yoga styles, session frequencies, and overall intervention durations. This lack of standardization makes it challenging to isolate the specific active therapeutic components or to establish an evidence-based dose–response guideline for clinical application.

Third, due to the physical and experiential nature of mind–body interventions, proper double-blinding is practically unfeasible. The lack of blinding among participants and instructors inevitably introduces potential expectation bias and placebo effects, which may artificially inflate self-reported psychological and behavioral outcomes.

Finally, as the application of yoga for digital addiction is a relatively nascent research field, it is highly susceptible to potential publication bias. Studies yielding positive, statistically significant outcomes are disproportionately more likely to be published, whereas null or negative findings may remain underreported. Acknowledging these methodological constraints is crucial, as they underscore the urgent need for more rigorous, large-scale, and actively controlled trial designs in future research to validate these preliminary findings.

## 6 Practical recommendations and future directions: an integrated socio-ecological framework

We propose a collaborative framework based on the Social-Ecological Model (SEM) (Bronfenbrenner, 1979), integrating “Home-School-Community” efforts to facilitate multi-level ecological synergy.

### 6.1 Reshaping the microsystem: family as the Core support unit

The family represents the most critical microsystem influencing adolescent behavior. Future interventions should aim to transform the family from a passive observer to an active agent of change. Given that parental support is a key determinant of adolescents’ adherence to health behaviors (Kwon et al., 2016), we advocate for shifting the clinical paradigm from “treating the adolescent” to “treating the family unit” through “Parent–Child Yoga” models.

Joint participation in yoga serves a dual function: it provides physical exercise while acting as a medium for emotional scaffolding. To overcome initial resistance and ensure feasibility, family-based protocols should start with brief sessions (e.g., 15–20 min) focusing on interactive, partner-based Asanas. This approach minimizes the perception of yoga as a rigid clinical treatment and instead frames it as a shared recreational activity, thereby fulfilling the adolescent’s basic psychological needs for relatedness and reducing the motivation to seek emotional compensation through virtual social interactions (Yang et al., 2022).

### 6.2 Strengthening the exosystem: clinical and educational integration

To support the microsystem, the outer layers of the social ecology—community medicine and school education—must provide structural reinforcement. Community healthcare providers should incorporate standardized screening (e.g., SAS-SV) into routine pediatric care. Clinicians can issue “Yoga practice “specifying FITT parameters (Frequency, Intensity, Time, Type) (Gupta, 2024). For adolescents with smartphone addiction, a feasible baseline FITT protocol would include: Frequency (3–4 days per week), Intensity (light-to-moderate, emphasizing parasympathetic activation), Time (20–30 min per session to align with adolescent attention spans), and Type (an integrated module of Pranayama and basic Asanas).

Schools offer the most accessible platform for large-scale implementation. Practically, this can be achieved by incorporating 10-min mindful breathing or simple postural stretches during morning assemblies or physical education warm-ups, significantly reducing barriers to entry. Capitalizing on yoga’s existing cultural acceptance as an extracurricular activity among Chinese students can significantly reduce resistance to participation and enhance long-term adherence to the intervention (Qin, 2022; Kerekes et al., 2024).

### 6.3 Managing adherence challenges in a digital age

A primary challenge in implementing mind–body interventions is maintaining adolescent adherence, particularly given the contrast between the slow pace of yoga and the rapid, high-dopamine feedback of smartphone use. Adolescents may initially find the stillness of meditation frustrating. To mitigate this, interventions should be framed as a positive tool for self-mastery rather than a punishment for “screen time.” Additionally, utilizing digital scaffolds—such as wearable devices that gamify heart rate variability (HRV) biofeedback—can help bridge the gap. By translating abstract physiological states into tangible digital rewards, these tools leverage familiar game mechanics to train parasympathetic dominance, gradually building offline mind–body awareness and improving long-term adherence (Lehrer et al., 2020).

### 6.4 Future research directions

While current translational evidence is promising, future research must prioritize rigorous, longitudinal Randomized Controlled Trials (RCTs) with larger sample sizes. Crucially, incorporating functional neuroimaging (e.g., fMRI) and Ecological Momentary Assessment (EMA) will be essential to empirically validate the proposed top-down and bottom-up neurobiological mechanisms, and to establish standardized, dose–response protocols tailored specifically for the neurodevelopmental window of adolescents.

## 7 Conclusion

The neuro-adaptive crisis precipitated by the digital era necessitates a systemic paradigm shift in intervention strategies. This review elucidates yoga’s potential as a precision behavioral medicine tool, providing a theoretical neurobiological rationale for its use in treating adolescent smartphone addiction. Mechanistically, we hypothesize that yoga may facilitate recovery through a dual framework: a potential bottom-up restoration of autonomic homeostasis (attenuating HPA axis and amygdala reactivity) and a proposed top-down enhancement of prefrontal inhibitory control (modulating dopaminergic/GABAergic dynamics), potentially supported by BDNF-mediated neuroplasticity.

However, neuroplasticity does not occur in a vacuum. Sustainable recovery relies on the construction of a holistic Social-Ecological Model that integrates the “Home-School-Community” triad. Future interventions will be most effective when they combine medical guidance with active family participation and consistent school-based support. Only through this synergistic approach can we cultivate true “Cognitive Resilience” in adolescents, empowering them to reclaim psychophysical autonomy in an increasingly digitalized world.
